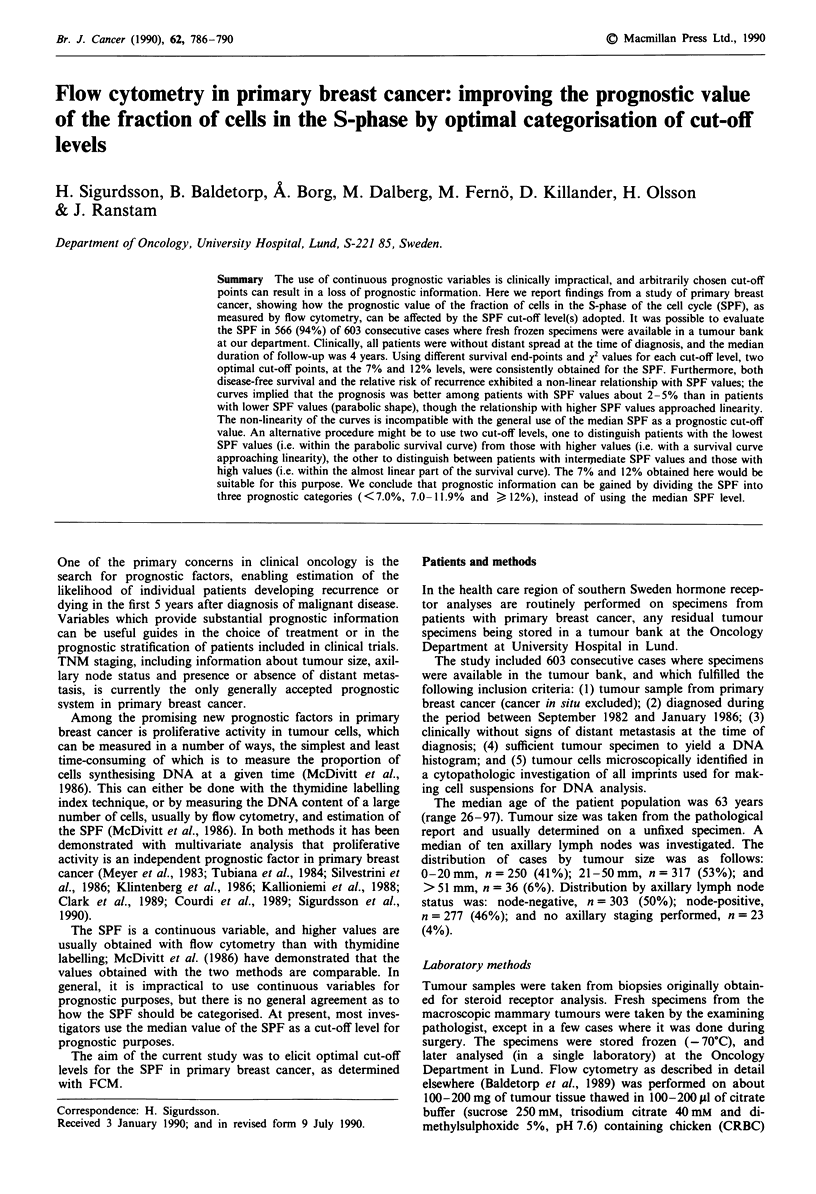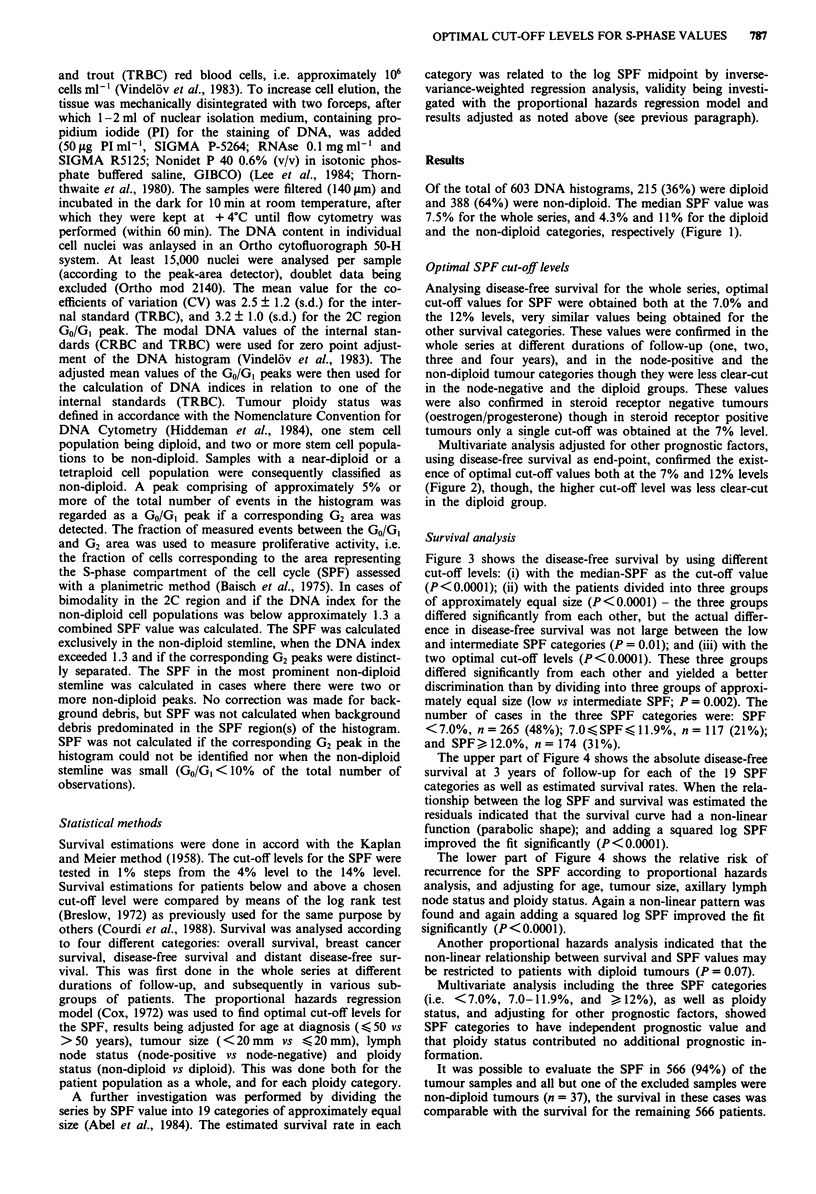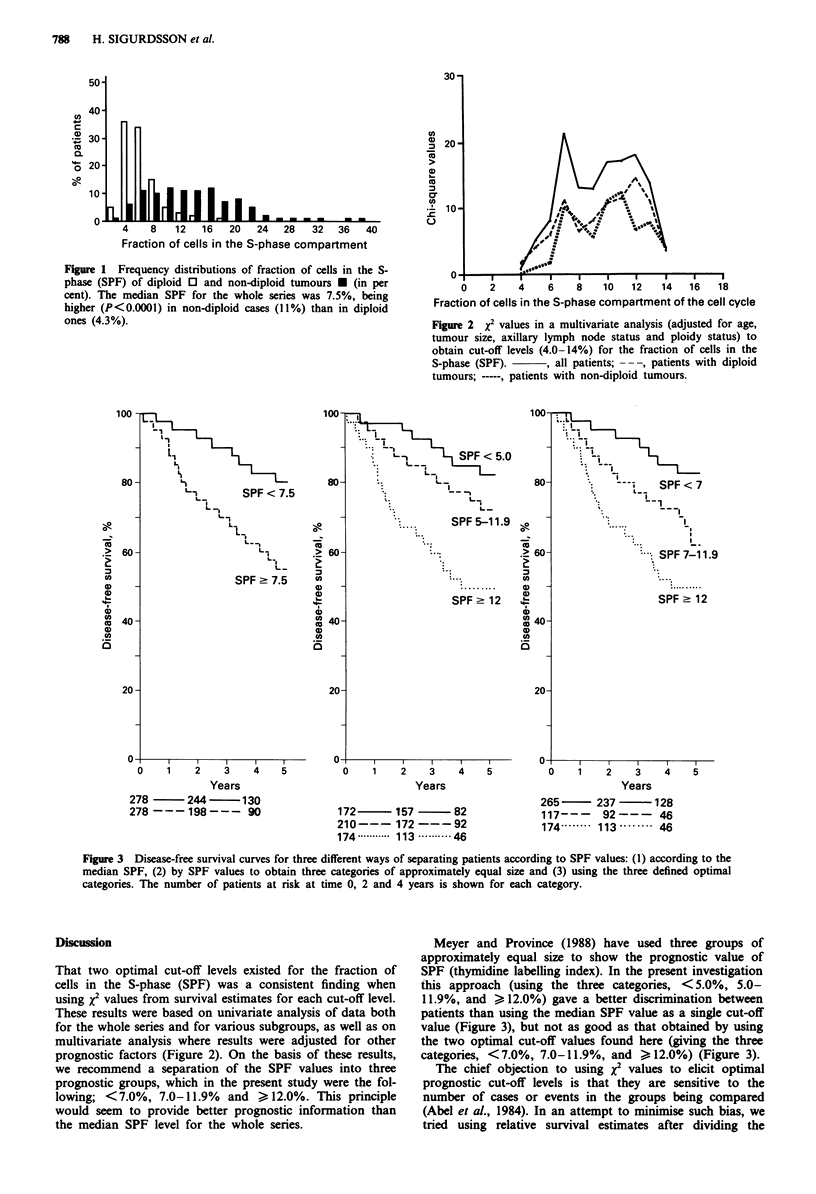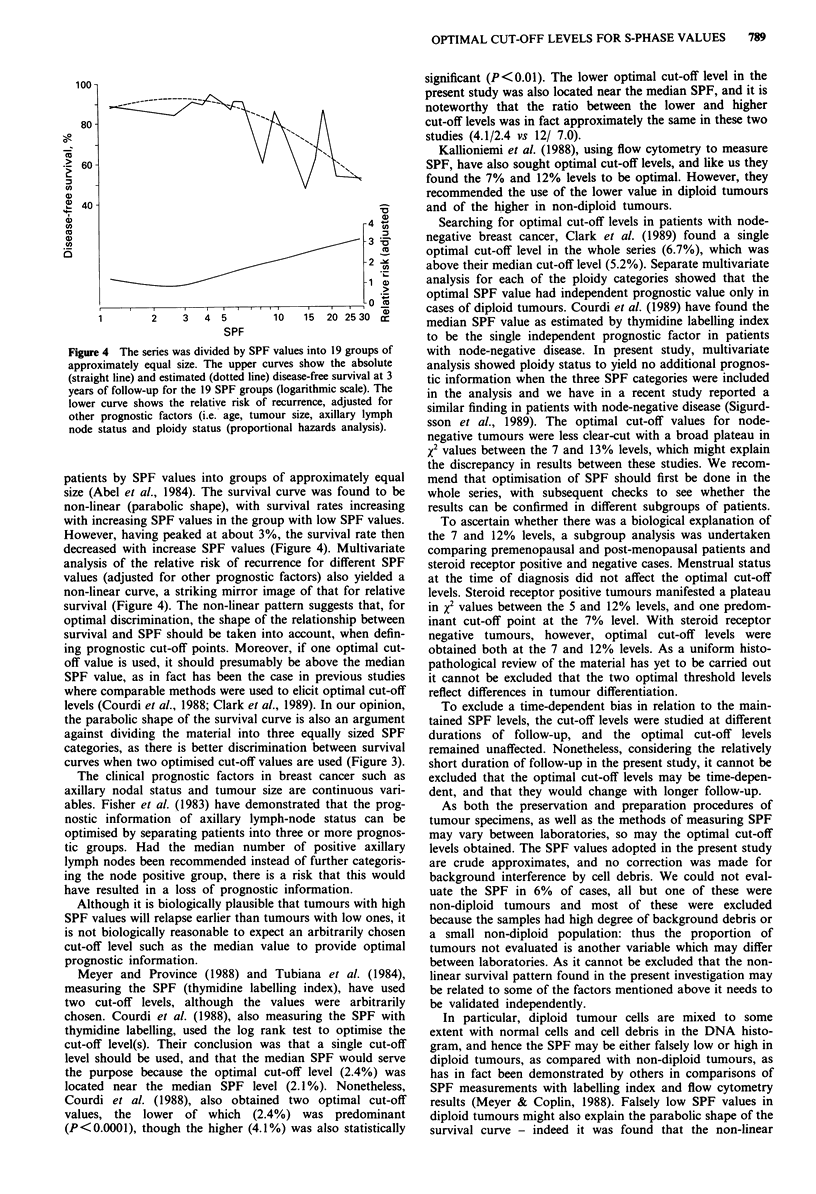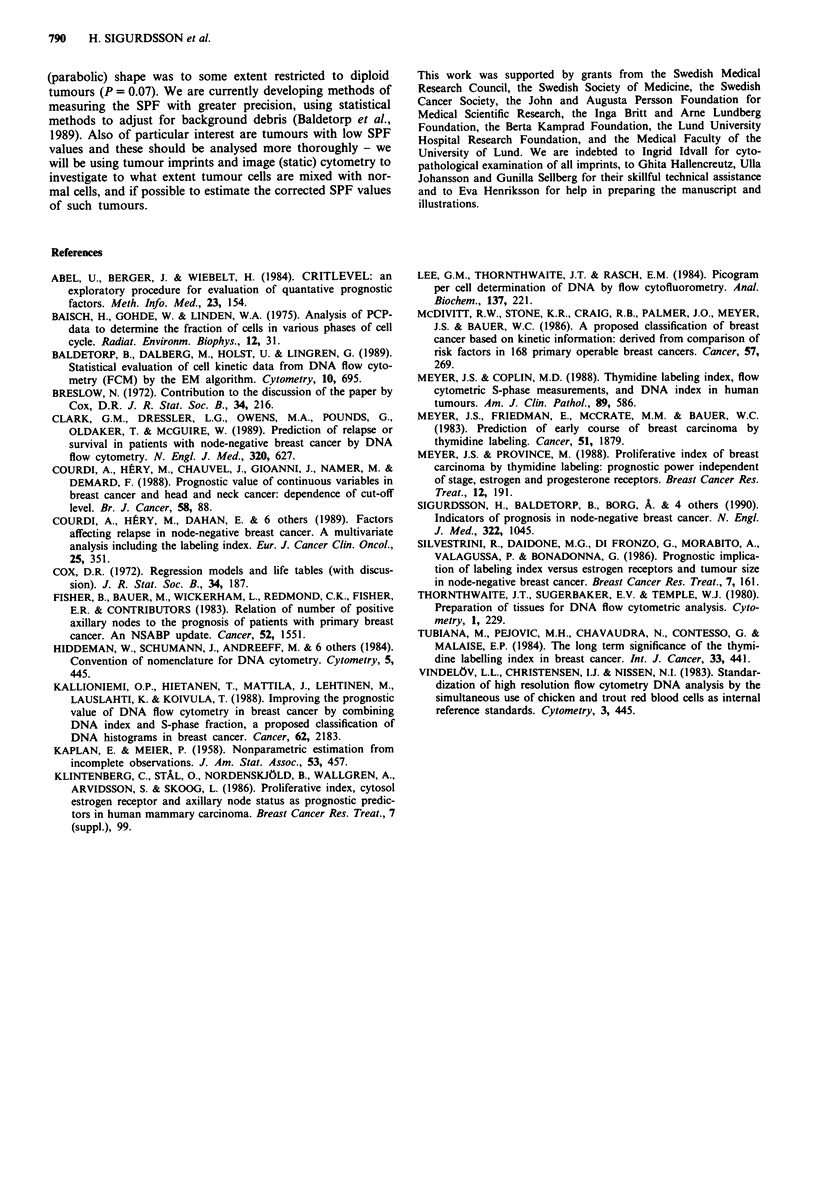# Flow cytometry in primary breast cancer: improving the prognostic value of the fraction of cells in the S-phase by optimal categorisation of cut-off levels.

**DOI:** 10.1038/bjc.1990.380

**Published:** 1990-11

**Authors:** H. Sigurdsson, B. Baldetorp, A. Borg, M. Dalberg, M. Fernö, D. Killander, H. Olsson, J. Ranstam

**Affiliations:** Department of Oncology, University Hospital, Lund, Sweden.

## Abstract

The use of continuous prognostic variables is clinically impractical, and arbitrarily chosen cut-off points can result in a loss of prognostic information. Here we report findings from a study of primary breast cancer, showing how the prognostic value of the fraction of cells in the S-phase of the cell cycle (SPF), as measured by flow cytometry, can be affected by the SPF cut-off level(s) adopted. It was possible to evaluate the SPF in 566 (94%) of 603 consecutive cases where fresh frozen specimens were available in a tumour bank at our department. Clinically, all patients were without distant spread at the time of diagnosis, and the median duration of follow-up was 4 years. Using different survival end-points and chi 2 values for each cut-off level, two optimal cut-off points, at the 7% and 12% levels, were consistently obtained for the SPF. Furthermore, both disease-free survival and the relative risk of recurrence exhibited a non-linear relationship with SPF values; the curves implied that the prognosis was better among patients with SPF values about 2-5% than in patients with lower SPF values (parabolic shape), though the relationship with higher SPF values approached linearity. The non-linearity of the curves is incompatible with the general use of the median SPF as a prognostic cut-off value. An alternative procedure might be to use two cut-off levels, one to distinguish patients with the lowest SPF values (i.e. within the parabolic survival curve) from those with higher values (i.e. with a survival curve approaching linearity), the other to distinguish between patients with intermediate SPF values and those with high values (i.e. within the almost linear part of the survival curve). The 7% and 12% obtained here would be suitable for this purpose. We conclude that prognostic information can be gained by dividing the SPF into three prognostic categories (less than 7.0%, 7.0-11.9% and greater than or equal to 12%), instead of using the median SPF level.


*Br. J. Cancer* (1990), **62**, 786-790 © Macmillan Press Ltd., 1990

### Flow cytometry in primary breast cancer: improving the prognostic value of the fraction of cells in the S-phase by optimal categorisation of cut-off levels

H. Sigurdsson, B. Baldetorp, Å. Borg, M. Dalberg, M. Fernö, D. Killander, H. Olsson & J. Ranstam

*Department of Oncology, University Hospital, Lund, S-221 85, Sweden.*

**Summary** The use of continuous prognostic variables is clinically impractical, and arbitrarily chosen cut-off points can result in a loss of prognostic information. Here we report findings from a study of primary breast cancer, showing how the prognostic value of the fraction of cells in the S-phase of the cell cycle (SPF), as measured by flow cytometry, can be affected by the SPF cut-off level(s) adopted. It was possible to evaluate the SPF in 566 (94%) of 603 consecutive cases where fresh frozen specimens were available in a tumour bank at our department. Clinically, all patients were without distant spread at the time of diagnosis, and the median duration of follow-up was 4 years. Using different survival end-points and $\chi^2$ values for each cut-off level, two optimal cut-off points, at the 7% and 12% levels, were consistently obtained for the SPF. Furthermore, both disease-free survival and the relative risk of recurrence exhibited a non-linear relationship with SPF values; the curves implied that the prognosis was better among patients with SPF values about 2-5% than in patients with lower SPF values (parabolic shape), though the relationship with higher SPF values approached linearity. The non-linearity of the curves is incompatible with the general use of the median SPF as a prognostic cut-off value. An alternative procedure might be to use two cut-off levels, one to distinguish patients with the lowest SPF values (i.e. within the parabolic survival curve) from those with higher values (i.e. with a survival curve approaching linearity), the other to distinguish between patients with intermediate SPF values and those with high values (i.e. within the almost linear part of the survival curve). The 7% and 12% obtained here would be suitable for this purpose. We conclude that prognostic information can be gained by dividing the SPF into three prognostic categories (<7.0%, 7.0-11.9% and ⩾12%), instead of using the median SPF level.

One of the primary concerns in clinical oncology is the search for prognostic factors, enabling estimation of the likelihood of individual patients developing recurrence or dying in the first 5 years after diagnosis of malignant disease. Variables which provide substantial prognostic information can be useful guides in the choice of treatment or in the prognostic stratification of patients included in clinical trials. TNM staging, including information about tumour size, axillary node status and presence or absence of distant metastasis, is currently the only generally accepted prognostic system in primary breast cancer.

Among the promising new prognostic factors in primary breast cancer is proliferative activity in tumour cells, which can be measured in a number of ways, the simplest and least time-consuming of which is to measure the proportion of cells synthesising DNA at a given time (McDivitt *et al.*, 1986). This can either be done with the thymidine labelling index technique, or by measuring the DNA content of a large number of cells, usually by flow cytometry, and estimation of the SPF (McDivitt *et al.*, 1986). In both methods it has been demonstrated with multivariate analysis that proliferative activity is an independent prognostic factor in primary breast cancer (Meyer *et al.*, 1983; Tubiana *et al.*, 1984; Silvestrini *et al.*, 1986; Klintenberg *et al.*, 1986; Kallioniemi *et al.*, 1988; Clark *et al.*, 1989; Courdi *et al.*, 1989; Sigurdsson *et al.*, 1990).

The SPF is a continuous variable, and higher values are usually obtained with flow cytometry than with thymidine labelling; McDivitt *et al.* (1986) have demonstrated that the values obtained with the two methods are comparable. In general, it is impractical to use continuous variables for prognostic purposes, but there is no general agreement as to how the SPF should be categorised. At present, most investigators use the median value of the SPF as a cut-off level for prognostic purposes.

The aim of the current study was to elicit optimal cut-off levels for the SPF in primary breast cancer, as determined with FCM.

Correspondence: H. Sigurdsson.
Received 3 January 1990; and in revised form 9 July 1990.

### Patients and methods

In the health care region of southern Sweden hormone receptor analyses are routinely performed on specimens from patients with primary breast cancer, any residual tumour specimens being stored in a tumour bank at the Oncology Department at University Hospital in Lund.

The study included 603 consecutive cases where specimens were available in the tumour bank, and which fulfilled the following inclusion criteria: (1) tumour sample from primary breast cancer (cancer *in situ* excluded); (2) diagnosed during the period between September 1982 and January 1986; (3) clinically without signs of distant metastasis at the time of diagnosis; (4) sufficient tumour specimen to yield a DNA histogram; and (5) tumour cells microscopically identified in a cytopathologic investigation of all imprints used for making cell suspensions for DNA analysis.

The median age of the patient population was 63 years (range 26-97). Tumour size was taken from the pathological report and usually determined on a unfixed specimen. A median of ten axillary lymph nodes was investigated. The distribution of cases by tumour size was as follows: 0-20 mm, $n = 250$ (41%); 21-50 mm, $n = 317$ (53%); and >51 mm, $n = 36$ (6%). Distribution by axillary lymph node status was: node-negative, $n = 303$ (50%); node-positive, $n = 277$ (46%); and no axillary staging performed, $n = 23$ (4%).

### *Laboratory methods*

Tumour samples were taken from biopsies originally obtained for steroid receptor analysis. Fresh specimens from the macroscopic mammary tumours were taken by the examining pathologist, except in a few cases where it was done during surgery. The specimens were stored frozen (−70°C), and later analysed (in a single laboratory) at the Oncology Department in Lund. Flow cytometry as described in detail elsewhere (Baldetorp *et al.*, 1989) was performed on about 100-200 mg of tumour tissue thawed in 100-200 µl of citrate buffer (sucrose 250 mM, trisodium citrate 40 mM and dimethylsulphoxide 5%, pH 7.6) containing chicken (CRBC)

and trout (TRBC) red blood cells, i.e. approximately $10^6$ cells $ml^{-1}$ (Vindelöv *et al.*, 1983). To increase cell elution, the tissue was mechanically disintegrated with two forceps, after which 1–2 ml of nuclear isolation medium, containing propidium iodide (PI) for the staining of DNA, was added (50 μg PI $ml^{-1}$, SIGMA P-5264; RNAse 0.1 mg $ml^{-1}$ and SIGMA R5125; Nonidet P 40 0.6% (v/v) in isotonic phosphate buffered saline, GIBCO) (Lee *et al.*, 1984; Thornthwaite *et al.*, 1980). The samples were filtered (140 μm) and incubated in the dark for 10 min at room temperature, after which they were kept at +4°C until flow cytometry was performed (within 60 min). The DNA content in individual cell nuclei was anlaysed in an Ortho cytofluorograph 50-H system. At least 15,000 nuclei were analysed per sample (according to the peak-area detector), doublet data being excluded (Ortho mod 2140). The mean value for the coefficients of variation (CV) was 2.5 ± 1.2 (s.d.) for the internal standard (TRBC), and 3.2 ± 1.0 (s.d.) for the 2C region $G_0/G_1$ peak. The modal DNA values of the internal standards (CRBC and TRBC) were used for zero point adjustment of the DNA histogram (Vindelöv *et al.*, 1983). The adjusted mean values of the $G_0/G_1$ peaks were then used for the calculation of DNA indices in relation to one of the internal standards (TRBC). Tumour ploidy status was defined in accordance with the Nomenclature Convention for DNA Cytometry (Hiddeman *et al.*, 1984), one stem cell population being diploid, and two or more stem cell populations to be non-diploid. Samples with a near-diploid or a tetraploid cell population were consequently classified as non-diploid. A peak comprising of approximately 5% or more of the total number of events in the histogram was regarded as a $G_0/G_1$ peak if a corresponding $G_2$ area was detected. The fraction of measured events between the $G_0/G_1$ and $G_2$ area was used to measure proliferative activity, i.e. the fraction of cells corresponding to the area representing the S-phase compartment of the cell cycle (SPF) assessed with a planimetric method (Baisch *et al.*, 1975). In cases of bimodality in the 2C region and if the DNA index for the non-diploid cell populations was below approximately 1.3 a combined SPF value was calculated. The SPF was calculated exclusively in the non-diploid stemline, when the DNA index exceeded 1.3 and if the corresponding $G_2$ peaks were distinctly separated. The SPF in the most prominent non-diploid stemline was calculated in cases where there were two or more non-diploid peaks. No correction was made for background debris, but SPF was not calculated when background debris predominated in the SPF region(s) of the histogram. SPF was not calculated if the corresponding $G_2$ peak in the histogram could not be identified nor when the non-diploid stemline was small ($G_0/G_1 < 10\%$ of the total number of observations).

### *Statistical methods*

Survival estimations were done in accord with the Kaplan and Meier method (1958). The cut-off levels for the SPF were tested in 1% steps from the 4% level to the 14% level. Survival estimations for patients below and above a chosen cut-off level were compared by means of the log rank test (Breslow, 1972) as previously used for the same purpose by others (Courdi *et al.*, 1988). Survival was analysed according to four different categories: overall survival, breast cancer survival, disease-free survival and distant disease-free survival. This was first done in the whole series at different durations of follow-up, and subsequently in various subgroups of patients. The proportional hazards regression model (Cox, 1972) was used to find optimal cut-off levels for the SPF, results being adjusted for age at diagnosis ($\leqslant 50$ *vs* $>50$ years), tumour size ($<20$ mm *vs* $\leqslant 20$ mm), lymph node status (node-positive *vs* node-negative) and ploidy status (non-diploid *vs* diploid). This was done both for the patient population as a whole, and for each ploidy category.

A further investigation was performed by dividing the series by SPF value into 19 categories of approximately equal size (Abel *et al.*, 1984). The estimated survival rate in each category was related to the log SPF midpoint by inverse-variance-weighted regression analysis, validity being investigated with the proportional hazards regression model and results adjusted as noted above (see previous paragraph).

### Results

Of the total of 603 DNA histograms, 215 (36%) were diploid and 388 (64%) were non-diploid. The median SPF value was 7.5% for the whole series, and 4.3% and 11% for the diploid and the non-diploid categories, respectively (Figure 1).

### *Optimal SPF cut-off levels*

Analysing disease-free survival for the whole series, optimal cut-off values for SPF were obtained both at the 7.0% and the 12% levels, very similar values being obtained for the other survival categories. These values were confirmed in the whole series at different durations of follow-up (one, two, three and four years), and in the node-positive and the non-diploid tumour categories though they were less clear-cut in the node-negative and the diploid groups. These values were also confirmed in steroid receptor negative tumours (oestrogen/progesterone) though in steroid receptor positive tumours only a single cut-off was obtained at the 7% level.

Multivariate analysis adjusted for other prognostic factors, using disease-free survival as end-point, confirmed the existence of optimal cut-off values both at the 7% and 12% levels (Figure 2), though, the higher cut-off level was less clear-cut in the diploid group.

### *Survival analysis*

Figure 3 shows the disease-free survival by using different cut-off levels: (i) with the median-SPF as the cut-off value ($P<0.0001$); (ii) with the patients divided into three groups of approximately equal size ($P<0.0001$) – the three groups differed significantly from each other, but the actual difference in disease-free survival was not large between the low and intermediate SPF categories ($P=0.01$); and (iii) with the two optimal cut-off levels ($P<0.0001$). These three groups differed significantly from each other and yielded a better discrimination than by dividing into three groups of approximately equal size (low *vs* intermediate SPF; $P=0.002$). The number of cases in the three SPF categories were: SPF $<7.0\%$, $n=265$ (48%); $7.0 \leqslant SPF \leqslant 11.9\%$, $n=117$ (21%); and SPF $\geqslant 12.0\%$, $n=174$ (31%).

The upper part of Figure 4 shows the absolute disease-free survival at 3 years of follow-up for each of the 19 SPF categories as well as estimated survival rates. When the relationship between the log SPF and survival was estimated the residuals indicated that the survival curve had a non-linear function (parabolic shape); and adding a squared log SPF improved the fit significantly ($P<0.0001$).

The lower part of Figure 4 shows the relative risk of recurrence for the SPF according to proportional hazards analysis, and adjusting for age, tumour size, axillary lymph node status and ploidy status. Again a non-linear pattern was found and again adding a squared log SPF improved the fit significantly ($P<0.0001$).

Another proportional hazards analysis indicated that the non-linear relationship between survival and SPF values may be restricted to patients with diploid tumours ($P=0.07$).

Multivariate analysis including the three SPF categories (i.e. $<7.0\%$, 7.0–11.9%, and $\geqslant 12\%$), as well as ploidy status, and adjusting for other prognostic factors, showed SPF categories to have independent prognostic value and that ploidy status contributed no additional prognostic information.

It was possible to evaluate the SPF in 566 (94%) of the tumour samples and all but one of the excluded samples were non-diploid tumours ($n=37$), the survival in these cases was comparable with the survival for the remaining 566 patients.

**Figure 1** Frequency distributions of fraction of cells in the S-phase (SPF) of diploid □ and non-diploid tumours ■ (in per cent). The median SPF for the whole series was 7.5%, being higher ($P<0.0001$) in non-diploid cases (11%) than in diploid ones (4.3%).

**Figure 2** $\chi^2$ values in a multivariate analysis (adjusted for age, tumour size, axillary lymph node status and ploidy status) to obtain cut-off levels (4.0–14%) for the fraction of cells in the S-phase (SPF). ———, all patients; – – –, patients with diploid tumours; -----, patients with non-diploid tumours.

**Figure 3** Disease-free survival curves for three different ways of separating patients according to SPF values: (1) according to the median SPF, (2) by SPF values to obtain three categories of approximately equal size and (3) using the three defined optimal categories. The number of patients at risk at time 0, 2 and 4 years is shown for each category.

### Discussion

That two optimal cut-off levels existed for the fraction of cells in the S-phase (SPF) was a consistent finding when using $\chi^2$ values from survival estimates for each cut-off level. These results were based on univariate analysis of data both for the whole series and for various subgroups, as well as on multivariate analysis where results were adjusted for other prognostic factors (Figure 2). On the basis of these results, we recommend a separation of the SPF values into three prognostic groups, which in the present study were the following; $<7.0\%$, 7.0–11.9% and $\geq 12.0\%$. This principle would seem to provide better prognostic information than the median SPF level for the whole series.

Meyer and Province (1988) have used three groups of approximately equal size to show the prognostic value of SPF (thymidine labelling index). In the present investigation this approach (using the three categories, $<5.0\%$, 5.0–11.9%, and $\geq 12.0\%$) gave a better discrimination between patients than using the median SPF value as a single cut-off value (Figure 3), but not as good as that obtained by using the two optimal cut-off values found here (giving the three categories, $<7.0\%$, 7.0–11.9%, and $\geq 12.0\%$) (Figure 3).

The chief objection to using $\chi^2$ values to elicit optimal prognostic cut-off levels is that they are sensitive to the number of cases or events in the groups being compared (Abel *et al.*, 1984). In an attempt to minimise such bias, we tried using relative survival estimates after dividing the

**Figure 4** The series was divided by SPF values into 19 groups of approximately equal size. The upper curves show the absolute (straight line) and estimated (dotted line) disease-free survival at 3 years of follow-up for the 19 SPF groups (logarithmic scale). The lower curve shows the relative risk of recurrence, adjusted for other prognostic factors (i.e. age, tumour size, axillary lymph node status and ploidy status (proportional hazards analysis).

patients by SPF values into groups of approximately equal size (Abel *et al.*, 1984). The survival curve was found to be non-linear (parabolic shape), with survival rates increasing with increasing SPF values in the group with low SPF values. However, having peaked at about 3%, the survival rate then decreased with increase SPF values (Figure 4). Multivariate analysis of the relative risk of recurrence for different SPF values (adjusted for other prognostic factors) also yielded a non-linear curve, a striking mirror image of that for relative survival (Figure 4). The non-linear pattern suggests that, for optimal discrimination, the shape of the relationship between survival and SPF should be taken into account, when defining prognostic cut-off points. Moreover, if one optimal cut-off value is used, it should presumably be above the median SPF value, as in fact has been the case in previous studies where comparable methods were used to elicit optimal cut-off levels (Courdi *et al.*, 1988; Clark *et al.*, 1989). In our opinion, the parabolic shape of the survival curve is also an argument against dividing the material into three equally sized SPF categories, as there is better discrimination between survival curves when two optimised cut-off values are used (Figure 3).

The clinical prognostic factors in breast cancer such as axillary nodal status and tumour size are continuous variables. Fisher *et al.* (1983) have demonstrated that the prognostic information of axillary lymph-node status can be optimised by separating patients into three or more prognostic groups. Had the median number of positive axillary lymph nodes been recommended instead of further categorising the node positive group, there is a risk that this would have resulted in a loss of prognostic information.

Although it is biologically plausible that tumours with high SPF values will relapse earlier than tumours with low ones, it is not biologically reasonable to expect an arbitrarily chosen cut-off level such as the median value to provide optimal prognostic information.

Meyer and Province (1988) and Tubiana *et al.* (1984), measuring the SPF (thymidine labelling index), have used two cut-off levels, although the values were arbitrarily chosen. Courdi *et al.* (1988), also measuring the SPF with thymidine labelling, used the log rank test to optimise the cut-off level(s). Their conclusion was that a single cut-off level should be used, and that the median SPF would serve the purpose because the optimal cut-off level (2.4%) was located near the median SPF level (2.1%). Nonetheless, Courdi *et al.* (1988), also obtained two optimal cut-off values, the lower of which (2.4%) was predominant ($P<0.0001$), though the higher (4.1%) was also statistically significant ($P<0.01$). The lower optimal cut-off level in the present study was also located near the median SPF, and it is noteworthy that the ratio between the lower and higher cut-off levels was in fact approximately the same in these two studies (4.1/2.4 *vs* 12/ 7.0).

Kallioniemi *et al.* (1988), using flow cytometry to measure SPF, have also sought optimal cut-off levels, and like us they found the 7% and 12% levels to be optimal. However, they recommended the use of the lower value in diploid tumours and of the higher in non-diploid tumours.

Searching for optimal cut-off levels in patients with node-negative breast cancer, Clark *et al.* (1989) found a single optimal cut-off level in the whole series (6.7%), which was above their median cut-off level (5.2%). Separate multivariate analysis for each of the ploidy categories showed that the optimal SPF value had independent prognostic value only in cases of diploid tumours. Courdi *et al.* (1989) have found the median SPF value as estimated by thymidine labelling index to be the single independent prognostic factor in patients with node-negative disease. In present study, multivariate analysis showed ploidy status to yield no additional prognostic information when the three SPF categories were included in the analysis and we have in a recent study reported a similar finding in patients with node-negative disease (Sigurdsson *et al.*, 1989). The optimal cut-off values for node-negative tumours were less clear-cut with a broad plateau in $\chi^2$ values between the 7 and 13% levels, which might explain the discrepancy in results between these studies. We recommend that optimisation of SPF should first be done in the whole series, with subsequent checks to see whether the results can be confirmed in different subgroups of patients.

To ascertain whether there was a biological explanation of the 7 and 12% levels, a subgroup analysis was undertaken comparing premenopausal and post-menopausal patients and steroid receptor positive and negative cases. Menstrual status at the time of diagnosis did not affect the optimal cut-off levels. Steroid receptor positive tumours manifested a plateau in $\chi^2$ values between the 5 and 12% levels, and one predominant cut-off point at the 7% level. With steroid receptor negative tumours, however, optimal cut-off levels were obtained both at the 7 and 12% levels. As a uniform histopathological review of the material has yet to be carried out it cannot be excluded that the two optimal threshold levels reflect differences in tumour differentiation.

To exclude a time-dependent bias in relation to the maintained SPF levels, the cut-off levels were studied at different durations of follow-up, and the optimal cut-off levels remained unaffected. Nonetheless, considering the relatively short duration of follow-up in the present study, it cannot be excluded that the optimal cut-off levels may be time-dependent, and that they would change with longer follow-up.

As both the preservation and preparation procedures of tumour specimens, as well as the methods of measuring SPF may vary between laboratories, so may the optimal cut-off levels obtained. The SPF values adopted in the present study are crude approximates, and no correction was made for background interference by cell debris. We could not evaluate the SPF in 6% of cases, all but one of these were non-diploid tumours and most of these were excluded because the samples had high degree of background debris or a small non-diploid population: thus the proportion of tumours not evaluated is another variable which may differ between laboratories. As it cannot be excluded that the non-linear survival pattern found in the present investigation may be related to some of the factors mentioned above it needs to be validated independently.

In particular, diploid tumour cells are mixed to some extent with normal cells and cell debris in the DNA histogram, and hence the SPF may be either falsely low or high in diploid tumours, as compared with non-diploid tumours, as has in fact been demonstrated by others in comparisons of SPF measurements with labelling index and flow cytometry results (Meyer & Coplin, 1988). Falsely low SPF values in diploid tumours might also explain the parabolic shape of the survival curve – indeed it was found that the non-linear

(parabolic) shape was to some extent restricted to diploid tumours ($P = 0.07$). We are currently developing methods of measuring the SPF with greater precision, using statistical methods to adjust for background debris (Baldetorp et al., 1989). Also of particular interest are tumours with low SPF values and these should be analysed more thoroughly – we will be using tumour imprints and image (static) cytometry to investigate to what extent tumour cells are mixed with normal cells, and if possible to estimate the corrected SPF values of such tumours.

This work was supported by grants from the Swedish Medical Research Council, the Swedish Society of Medicine, the Swedish Cancer Society, the John and Augusta Persson Foundation for Medical Scientific Research, the Inga Britt and Arne Lundberg Foundation, the Berta Kamprad Foundation, the Lund University Hospital Research Foundation, and the Medical Faculty of the University of Lund. We are indebted to Ingrid Idvall for cytopathological examination of all imprints, to Ghita Hallencreutz, Ulla Johansson and Gunilla Sellberg for their skillful technical assistance and to Eva Henriksson for help in preparing the manuscript and illustrations.

### References

ABEL, U., BERGER, J. & WIEBELT, H. (1984). CRITLEVEL: an exploratory procedure for evaluation of quantative prognostic factors. *Meth. Info. Med.*, **23**, 154.

BAISCH, H., GOHDE, W. & LINDEN, W.A. (1975). Analysis of PCP-data to determine the fraction of cells in various phases of cell cycle. *Radiat. Environm. Biophys.*, **12**, 31.

BALDETORP, B., DALBERG, M., HOLST, U. & LINGREN, G. (1989). Statistical evaluation of cell kinetic data from DNA flow cytometry (FCM) by the EM algorithm. *Cytometry*, **10**, 695.

BRESLOW, N. (1972). Contribution to the discussion of the paper by Cox, D.R. *J. R. Stat. Soc. B.*, **34**, 216.

CLARK, G.M., DRESSLER, L.G., OWENS, M.A., POUNDS, G., OLDAKER, T. & MCGUIRE, W. (1989). Prediction of relapse or survival in patients with node-negative breast cancer by DNA flow cytometry. *N. Engl. J. Med.*, **320**, 627.

COURDI, A., HÉRY, M., CHAUVEL, J., GIOANNI, J., NAMER, M. & DEMARD, F. (1988). Prognostic value of continuous variables in breast cancer and head and neck cancer: dependence of cut-off level. *Br. J. Cancer*, **58**, 88.

COURDI, A., HÉRY, M., DAHAN, E. & 6 others (1989). Factors affecting relapse in node-negative breast cancer. A multivariate analysis including the labeling index. *Eur. J. Cancer Clin. Oncol.*, **25**, 351.

COX, D.R. (1972). Regression models and life tables (with discussion). *J. R. Stat. Soc. B.*, **34**, 187.

FISHER, B., BAUER, M., WICKERHAM, L., REDMOND, C.K., FISHER, E.R. & CONTRIBUTORS (1983). Relation of number of positive axillary nodes to the prognosis of patients with primary breast cancer. An NSABP update. *Cancer*, **52**, 1551.

HIDDEMAN, W., SCHUMANN, J., ANDREEFF, M. & 6 others (1984). Convention of nomenclature for DNA cytometry. *Cytometry*, **5**, 445.

KALLIONIEMI, O.P., HIETANEN, T., MATTILA, J., LEHTINEN, M., LAUSLAHTI, K. & KOIVULA, T. (1988). Improving the prognostic value of DNA flow cytometry in breast cancer by combining DNA index and S-phase fraction, a proposed classification of DNA histograms in breast cancer. *Cancer*, **62**, 2183.

KAPLAN, E. & MEIER, P. (1958). Nonparametric estimation from incomplete observations. *J. Am. Stat. Assoc.*, **53**, 457.

KLINTENBERG, C., STÅL, O., NORDENSKJÖLD, B., WALLGREN, A., ARVIDSSON, S. & SKOOG, L. (1986). Proliferative index, cytosol estrogen receptor and axillary node status as prognostic predictors in human mammary carcinoma. *Breast Cancer Res. Treat.*, **7** (suppl.), 99.

LEE, G.M., THORNTHWAITE, J.T. & RASCH, E.M. (1984). Picogram per cell determination of DNA by flow cytofluorometry. *Anal. Biochem.*, **137**, 221.

MCDIVITT, R.W., STONE, K.R., CRAIG, R.B., PALMER, J.O., MEYER, J.S. & BAUER, W.C. (1986). A proposed classification of breast cancer based on kinetic information: derived from comparison of risk factors in 168 primary operable breast cancers. *Cancer*, **57**, 269.

MEYER, J.S. & COPLIN, M.D. (1988). Thymidine labeling index, flow cytometric S-phase measurements, and DNA index in human tumours. *Am. J. Clin. Pathol.*, **89**, 586.

MEYER, J.S., FRIEDMAN, E., MCCRATE, M.M. & BAUER, W.C. (1983). Prediction of early course of breast carcinoma by thymidine labeling. *Cancer*, **51**, 1879.

MEYER, J.S. & PROVINCE, M. (1988). Proliferative index of breast carcinoma by thymidine labeling: prognostic power independent of stage, estrogen and progesterone receptors. *Breast Cancer Res. Treat.*, **12**, 191.

SIGURDSSON, H., BALDETORP, B., BORG, Å. & 4 others (1990). Indicators of prognosis in node-negative breast cancer. *N. Engl. J. Med.*, **322**, 1045.

SILVESTRINI, R., DAIDONE, M.G., DI FRONZO, G., MORABITO, A., VALAGUSSA, P. & BONADONNA, G. (1986). Prognostic implication of labeling index versus estrogen receptors and tumour size in node-negative breast cancer. *Breast Cancer Res. Treat.*, **7**, 161.

THORNTHWAITE, J.T., SUGERBAKER, E.V. & TEMPLE, W.J. (1980). Preparation of tissues for DNA flow cytometric analysis. *Cytometry*, **1**, 229.

TUBIANA, M., PEJOVIC, M.H., CHAVAUDRA, N., CONTESSO, G. & MALAISE, E.P. (1984). The long term significance of the thymidine labelling index in breast cancer. *Int. J. Cancer*, **33**, 441.

VINDELÖV, L.L., CHRISTENSEN, I.J. & NISSEN, N.I. (1983). Standardization of high resolution flow cytometry DNA analysis by the simultaneous use of chicken and trout red blood cells as internal reference standards. *Cytometry*, **3**, 445.